# Relationships between carbon fluxes and environmental factors in a drip-irrigated, film-mulched cotton field in arid region

**DOI:** 10.1371/journal.pone.0192467

**Published:** 2018-02-07

**Authors:** Xiaoyu Li, Lijuan Liu, Huijin Yang, Yan Li

**Affiliations:** 1 State Key Laboratory of Subtropical Silviculture, Zhejiang A&F University, Lin’an, China; 2 Xinjiang Institute of Ecology and Geography, Chinese Academy of Sciences, Xinjiang, China; 3 School of Remote Sensing and Information Engineering, Wuhan University, Wuhan, China; Pacific Northwest National Laboratory, UNITED STATES

## Abstract

Environmental factors and human activities play important roles in carbon fixation and emissions generated from croplands. Eddy covariance measurements in a drip-irrigated, film-mulched cotton field were used to analyze the relationships between carbon fluxes and environmental factors in Wulanwusu, northern Xinjiang, an arid region of Northwest China. Our results showed that the cumulative net carbon flux (NEE) was -304.8 g C m^-2^ (a strong sink) over the whole cotton growing season in 2012, which was more than that in cotton cropland without plastic film mulching and drip-irrigation. Moreover, when time is scaled up from a half-hour to a month, the correlations of gross primary production (GPP) to air temperature (T_air_), net solar radiation (R_n_) and soil water content (SWC) gradually become stronger due to ecosystem resistance and resilience as well as the protection of plastic film mulching. The GPP is more strongly correlated with R_n_ than T_air_ at time scales from minutes to days, while it reverses at time scales from days to weeks. This outcome is largely determined by the biochemical characteristics of photosynthesis. SWC and vapor pressure deficit (VPD) at all time scales are weakly correlated with GPP because plastic film mulching and regularly drip-irrigation allow soil to maintain sufficient water.

## Introduction

Carbon dioxide emissions generated from upland agro-ecosystems are a major source of atmospheric greenhouse gases [1]. Agricultural lands occupy 37% of the Earth’s land surface and account for 20% of atmospheric carbon dioxide [2]. Additionally, agricultural lands are a non-ignorable carbon sink. Conversely, an elevated concentration of atmosphere carbon dioxide could have a large impact on future agricultural productivity [3–5], as it can accelerate agricultural crop growth rates [6,7]. Consequently, measurements of carbon fluxes generated from agro-ecosystems in an arid region are essential to an analysis of carbon flux dynamics.

Agricultural lands are extremely important in oases of Xinjiang, an extensive arid region of Northwest China. Although oases account for only 4–5% of the total area of this region, over 90% of the province’s population and 95% of its wealth are concentrated within them [8]. The Manas River Watershed is a typical mountain-oasis-desert ecosystem in the arid region of Northwest China. The rapid growth of the regional population and the continuous expansion of agricultural lands in this watershed induce acute and frequent water shortages [8,9]. The agricultural water consumption currently accounts for 96.2% of the total water consumption in this watershed [8]. Therefore, water-saving irrigation is of great importance in this arid region. The intensifying human exploitation in this watershed is typical in the arid region of Northwest China [9,10].

Mulched-drip irrigation is a great approach to save water. Drip irrigation was combined with film mulching successfully in 1996 after the Eighth Agricultural Division of Xinjiang Production and Construction Corps, which is located in the Manas River Watershed, experimented with the combined technology in a small area. It has been widely promoted and applied since 1999, and now, the cropland covered by plastic film mulching with drip irrigation accounts for up to 80% of the total irrigated area in the Manas River Watershed. It has widely taken the place of the traditional furrow irrigation and is the predominant irrigation method in recent years in the most of China’s arid regions [11]. Additionally, the Manas River Watershed is the biggest cotton belt in Xinjiang, with a cotton-planting area of up to 52% of the agricultural land. Therefore, cotton fields in this watershed can be considered representative of the entire arid region, with the utilization of plastic film mulching and drip irrigation key issues.

Plastic film mulching with drip irrigation alters the soil microenvironment and has a great impact on the agro-ecosystem, bringing with it many advantages such as weed inhibition, soil temperature improvement, water evaporation reduction, soil organic carbon stock enhancement, greenhouse gas emission reductions and crop yield increase [12–16]. The impact of partial surface mulch on soil heat and water flow was examined by many studies [17–19]. More recently, some researchers have compared the effects of flood irrigation without film mulching and plastic film mulching with drip irrigation on carbon and water fluxes in arid or semi-arid regions [12, 14, 16, 20, 21]. Some studies have also shown that plastic film mulching with drip irrigation has great effects on nitrous oxide and methane emissions in arid regions [11, 13, 22, 23]. For example, Berger et al. (2013) [23] contended that polyethylene mulching may decrease nitrous oxide emissions. Additionally, there exist some studies concerning the relationships of carbon fluxes with environmental factors (R_n_, T_air_, VPD and SWC) [24–31]. Vourlitis et al (2000) [32] and Whitley et al. (2009) [33] discussed the responses of stand transpiration to R_n_, VPD and SWC at different time scales, and Liu et al. (2009) [21] analyzed the correlations between temperature and carbon fluxes in different types of film mulching. However, these studies have only concentrated on the influences of plastic film mulching with drip irrigation on soil water, greenhouse gas emission and crop yield, and so on; there is still a lack of information about the quantitative analyses of the relationships between carbon fluxes and environmental factors under the influence of plastic film mulching with drip irrigation at various time scales. Consequently, in a drip-irrigated, film-mulched cotton field, analysis of their relationships is essential to understanding the influence of environmental factors on carbon fluxes [5], and provides the scientific basis for emphasizing different environmental factors at different time scales.

## Materials and methods

### Site description

The study site (latitude 44°17′N, longitude 85°49′E, elevation 468.2 m) is located at the Wulanwusu Agrometeorological Experiment Station on the northern slope of the Tianshan Mountains in Xinjiang, Northwest China. This region is subject to a typical temperate continental climate. Using 30 years (1980–2010) of climatology data from a meteorological dataset, the precipitation shows large fluctuations, varying from 71.95 to 242.3 mm, with an average precipitation of 129.88 mm over the growing season. The mean length of the full growing season is approximately 169 days. The mean annual sunshine duration is 2861 hours per year with approximately 170 frost-free days [8]. The soil type is gray desert soil, consisting of 42% silt, 39% sand and 19% clay at 0–30 mm depth. The mean field moisture capacity is 27.1%, and the bulk density is approximately 1.3 g cm^-3^ [8]. Furthermore, the average T_air_ and precipitation was 22.42°C and 50.5 mm in the 2012 growing season.

The experimental plot at the station is 39600 m^2^ (300m×120m) and is flat enough for cotton with a mean population density of approximately 24 plants m^-2^. The cotton in the experimental plot was sowed on April 17 and harvested on September 23, 2012 (the entire growth period of cotton is 158 days). The field management of cotton was in accordance with local practices to ensure a realistic characterization of NEE and GPP from an agricultural area in this watershed; 80% of this plot is covered with plastic film mulching of 0.08 mm thickness; a drip hose was placed under the plastic film, an irrigation method referred to as ‘film-mulched drip irrigation’ [8]. Plastic film mulching is kept on the field from cotton sowing to harvesting.

### Eddy covariance flux measurement

An eddy covariance (EC) system is located in a cotton field with relatively homogeneous and adequate fetch. Data (including carbon fluxes, sensible heat fluxes and latent heat fluxes) were established in 2009. Data from 17 April 2012 to 23 September 2012 are used in this study. The site was equipped with a three-dimensional sonic anemometer (CSAT3, Campbell, USA) for measuring wind speed and wind direction and an open-path infrared CO2/H2O analyzer (IRGA, LI-7500, Li-Cor, USA) for measuring carbon dioxide and water vapor concentrations on a mast at a 4-m height. Furthermore, the flux mast was also equipped with some additional sensors. R_n_ is measured with a CNR1 (Kipp & Zonen,Netherlands), T_air_ and air humidity with a HMP45C (Vaisala, Helsinki, Finland), soil heat flux with two HFP01 sensors (Hukseflux, Netherlands) and soil temperature with four Thermocouples (TCAV, Campbell, USA). Data measured by these sensors can be recorded automatically at 10 Hz on a data logger (CR3000, Campbell, USA).

### Data processing

The eddy covariance method was applied during steady atmospheric conditions and on relatively flat terrain; otherwise, it would be vulnerable to systematic bias errors [34]. Therefore, we filtered some outliers and fill gaps to improve the quality of the trace gas fluxes.

Using the EddyPro software, these data were corrected by performing the following: three-axis coordinate rotations to eliminate errors due to sensor tilt relative to the terrain surface [35, 36]; WPL correction to compensate for the fluctuations of temperature and water vapor due to the presence of heat and water vapor flux [37,38]; and spectral corrections to compensate for the spectral attenuation due to the separation between sensors [39].

To minimize data errors, we screened the data, the process of which consisted of two steps. First, the outliers were detected by the comparison of half-hourly fluxes X_i_ with a 200-data point moving mean (X_gi_) and standard deviation (X_sdi_) [40]: if

$$X_{i}<X_{gi}‑(2.5\times X_{sdi})$$ (1)

or

$$X_{i}>X_{gi}+(2.5\times X_{sdi})$$ (2)

then X_i_ was screened from the dataset. In addition, the data of daytime and nighttime were screened separately. Nighttime was defined as a downward solar radiation < 20 Wm^-2^[41].

Second, nighttime NEE that were less than 0 and larger than 0.6 mg C m^-2^s^-1^werealso screened [42,43]. However, the condition of low turbulence at night [44] could have caused the nighttime fluxes to be underestimated [45–48]. Accordingly, if Ustar is less than 0.15 ms^-1^, as determined by an average values test (AVT) [43], then the NEE that corresponds to these Ustar are screened.

After screening, approximately 82% of the trace gas fluxes remained. However, these incomplete data were not enough for further analysis. Thus, to accurately calculate values of trace gas fluxes, gap-filling was imperative. In this study, we filled gaps using the online eddy covariance processing tool of the Department of Biogeochemical Integration at the Max Planck Institute for Biogeochemistry (http://www.bgc-jena.mpg.de/~MDIwork/eddyproc/).

### Flux-partitioning

NEE exchange is partitioned into two components, GPP and R_eco_ [28,47]:

$$NEE=R_{eco}‑GPP$$ (3)

The R_eco_ and NEE were calculated by the online eddy covariance processing tool of the Department of Biogeochemical Integration at the Max Planck Institute for Biogeochemistry (http://www.bgc-jena.mpg.de/~MDIwork/eddyproc/).

### Response functions of GPP

R_n_, T_air_, and SWC are the primary environmental factors that affect ecosystem carbon uptake and release[30,32,49].

We assessed the relationship between GPP and T_air_ using the nonlinear fitting function [50,51]:

$$y=a\times \exp(\;‑k_{1}\times {\left(x‑k_{3}\right)}^{2}/(x+k_{2}))$$ (4)

where y represents GPP; x is VPD or T_air_; a is the maximum value of GPP (GPP_max_); k_1_ and k_2_ represent the shape of the response curve; and k_3_ describes the value of the VPD or T_air_ at which GPP is maximized.

To study the responses of the GPP to R_n_ at the half-hourly time scale, we used the asymptote equation to obtain light–response curves [33,51]:

y=a×x/(x+b) (5)

where y represents GPP; x is R_n_; a is the light saturation GPP (GPP_max_); and b is the rate of change.

At half-monthly and monthly time scales, the responses of GPP to T_air_ and R_n_ are described using linear relationships with log-transformed data, ln(*y*) = a*x* + b, which is similar to the response of R_eco_ against T_soil_ [25].

### Data quality assessment

According to the first law of thermodynamics, the sum of the estimated latent and sensible heat flux must be equivalent to all other energy sinks and sources, and energy balance closure can be expressed as[42,52]

$$LE+H=R_{n}‑G‑S‑Q$$ (6)

where R_n_ is the net radiation, LE the latent heat flux, H the sensible heat flux, S canopy heat storage, G the soil heat flux, and Q the sum of all additional energy sources. Q is neglected as a small term. Canopy heat storage can also be neglected in short canopies on the condition of the vegetation height being less than 8 m[52].

**Fig 1:**
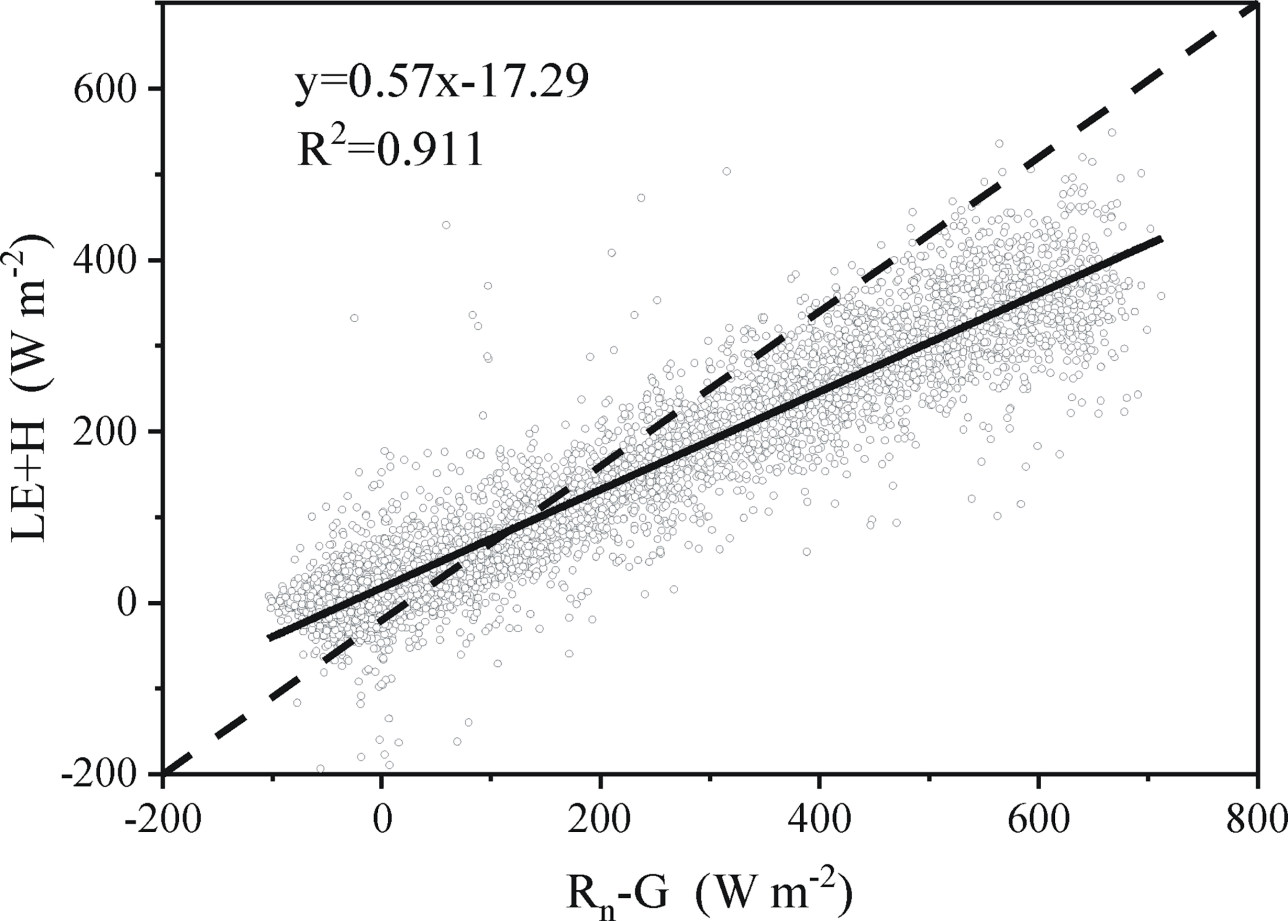
Energy-balance closure of cotton during entire growth period. **The slope of the solid line represents the degree of energy balance closure.** (R_n_ is the net radiation, G the soil heat flux, LE is the latent heat flux, H is the sensible heat flux).

The slope of LE+H against R_n_-G is 0.57 (Fig 1) over the entire growing season; the energy balance is not closed.

The data shown in all figures, and supporting all of the principal results in this paper, are publicly available:

(http://www.editorialmanager.com/pone/download.aspx?id = 21540143&guid = 5ccdf696-9d97-471f-b758-4704b73813ed&scheme = 1).

## Results

### Diurnal variations in NEE

**Fig 2:**
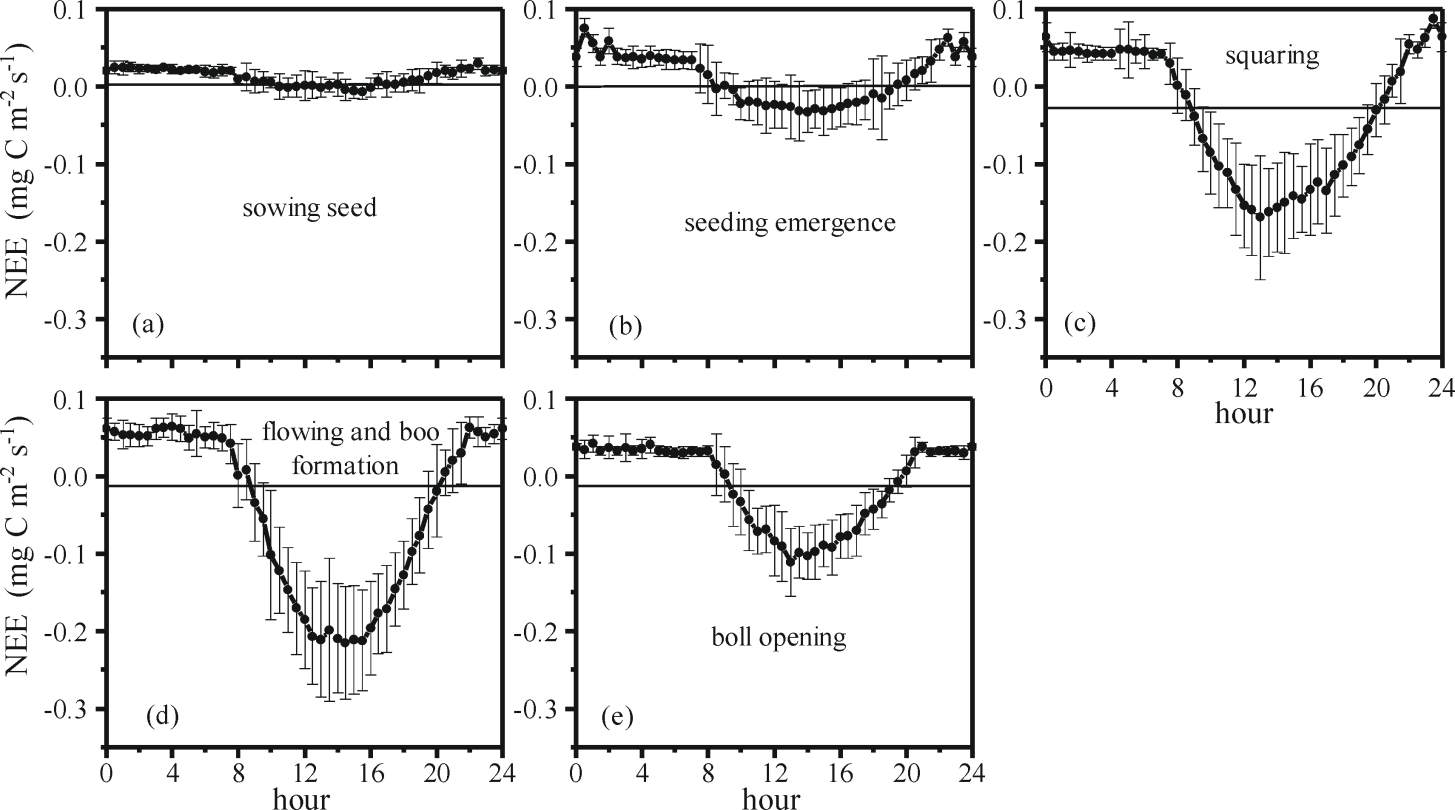
Average diurnal profiles of NEE (net ecosystem exchange) for five growth stages and mean values of the diurnal variation in NEE for each growth stages. The vertical bars represent the standard deviation of the mean values.

The mean diurnal variations in NEE during each of the growth stages of cotton are shown in Fig 2. We calculated the random uncertainty of each data point. For example, during the sowing period, the random uncertainty of NEE at 12:00 am represented the standard deviation of all of NEE at this time and growth stage; the uncertainty is equal to the error bars given in Fig 2.

Overall, the dynamic of NEE in all growing stages shows a single-peak curve. In the periods of seeding emergence and boll opening, NEE is positive at night due only to respiration and is negative in the day predominantly due to photosynthesis. The absolute value of NEE is largest at midday due to sufficient solar radiation and high temperatures.

NEE has a conspicuous distinction at different growth stages; the absolute value of NEE during the sowing period (Fig 2A) is the lowest of all five growth stages due to the absence of photosynthesis and depending on the respiration. The day length of carbon fixation is longest in the periods of squaring, flowering and boll formation (14 hours, Fig 2D). Carbon fixation in the squaring (Fig 2C) begins early in the morning (approximately at 8 am), but carbon fixation in seeding emergence is delayed by approximately 0.5 hours (Fig 2B).

### Carbon sink or source

**Fig 3:**
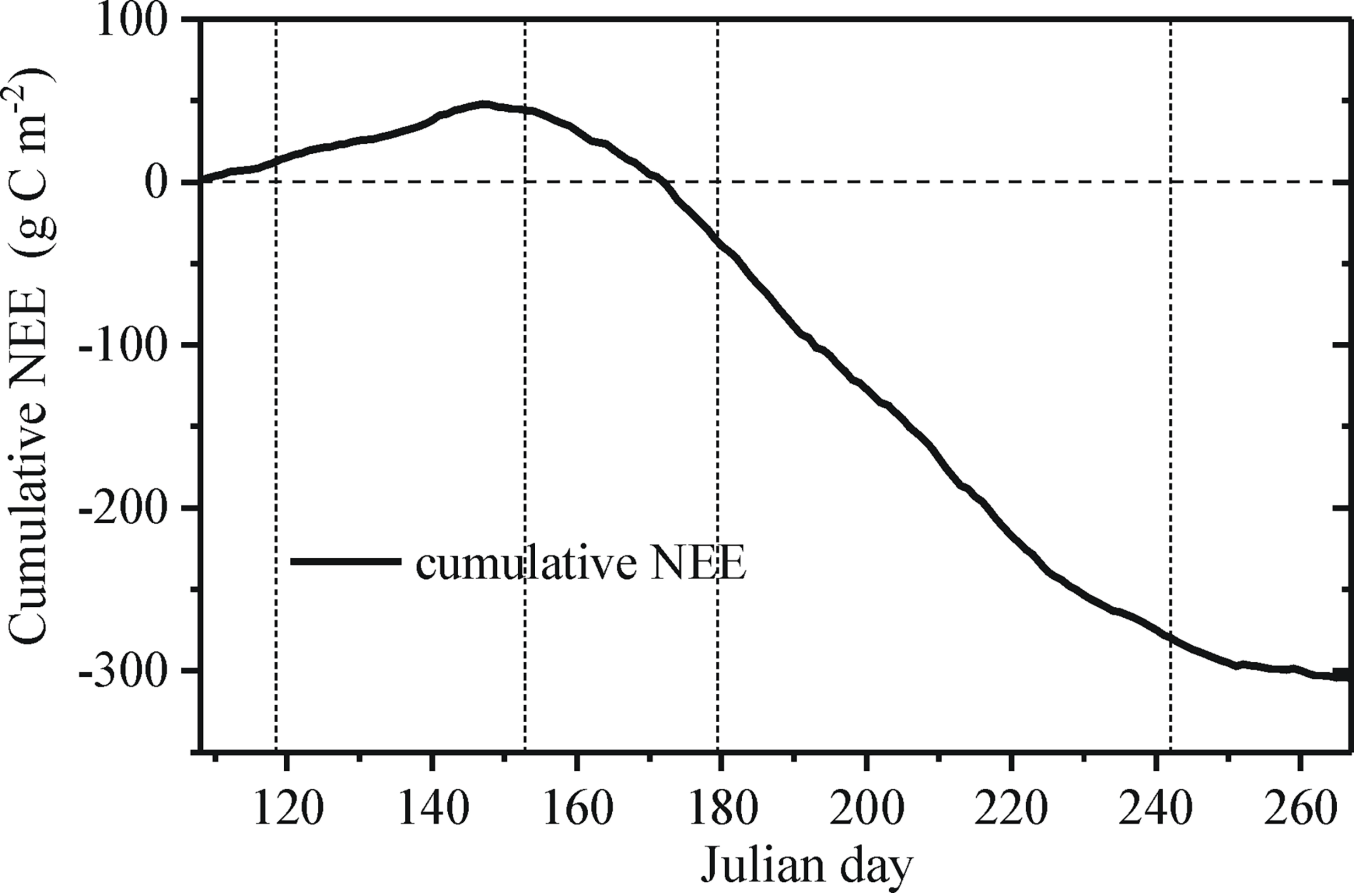
Cumulative time series of total daily NEE (net ecosystem exchange). The vertical dotted lines are the boundaries of different growing stages. The horizontal dotted line represents the zero reference.

**Fig 4:**
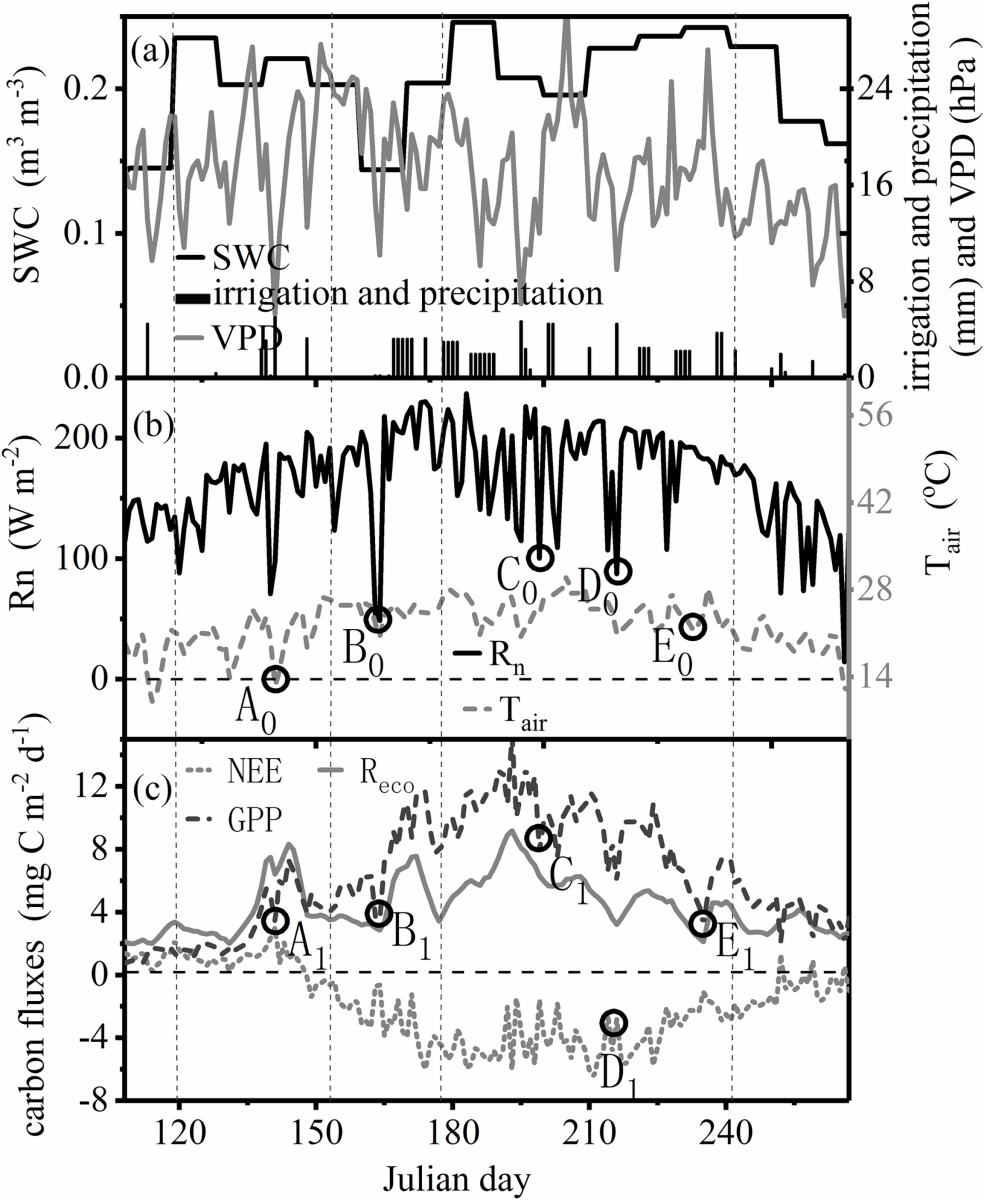
Total daily GPP (gross primary production) and mean daily T_air_ (air temperature), R_n_ (net solar radiation), rainfall, VPD (vapor pressure deficit) and SWC (soil water content). The vertical dotted lines are the boundaries of different growing stages. The horizontal dotted lines represent the zero reference.

The cotton field is a strong sink of carbon over the growing season as a whole, as illustrated in Fig 3. Over the entire growing season, cumulative NEE first increases and then decreases (Fig 3). From the beginning of sowing to the end of the seed emergence, NEE is positive, and the cotton field is a carbon source due to little photosynthesis and the predominance of soil respiration. The switch from source to sink is clearly evident around Julian day 148 (Fig 4C); carbon fixation then persists to the end of boll opening, the photosynthesis of cotton being dominant. Over the entire cotton growing season, the cumulative NEE is approximately -304.8 g C m^-2^.

### Variations in daily NEE, R_eco_ and GPP

Overall, the dynamic of NEE, ecosystem respiration (R_eco_) or GPP shows a single-peak curve in cotton’s entire development stage. Over the entire growing season, NEE, R_eco_ and GPP reach a maximum of -6.4 gCm^-2^d^-1^**,** 9.18 gC m^-2^d^-1^ and 15.19 gC m^-2^d^-1^, respectively. The flowering and boll formation period (days 180–242) accounts for 80.58%, 47.68% and 57.60% of the NEE, R_eco_ and GPP, respectively. Each of these percentages is highest during the growing season. Therefore, the fourth period is the primary growth stage. Additionally, the cumulative R_eco_ and GPP are approximately 706.18g C m^-2^and 1011.00 g C m^-2^, respectively.

Meteorological characteristics have obvious temporal variability during the growing season (Fig 4A and 4B). Fig 4 shows that the trend of GPP changes corresponding with the change of environmental factors (R_n_, T_air_ and VPD, etc.). For instance, irrigation of 4.5 mm on days 201–202 induces the increase of GPP by approximately 3.40%; the low points of R_n_ and T_air_ in the local region correspond with the local minimum GPP such as A_0_ and A_1_, B_0_ and B_1_, and C_0_ and C_1_. However, there is another situation: as R_n_ decreases, the GPP increases. Therefore, variability in GPP is related to environmental factors (R_n_, T_air_ and SWC) (Fig 4), which are important controlling factors of the carbon balance [53].

### Responses of GPP at half-hourly, daily and monthly time scales

At the half-hour time scale, it is assumed that the responses of GPP to each environmental factor is independent of the other variables when values for the other environmental factors are not limiting [54]. The data are selected for the daytime (downward solar radiation > 20 W m^-2^) of the entire growing season.

**Fig 5:**
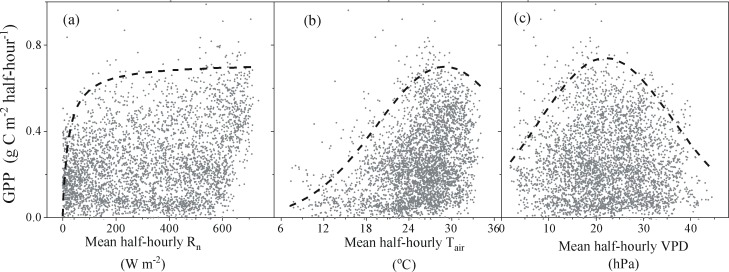
The response curves of total half-hourly GPP (gross primary production) to mean half-hourly R_n_ (net solar radiation) (a), T_air_ (air temperature) (b), and VPD (vapor pressure deficit) (c) at the 95% confidence interval.

As R_n_ increases, the GPP asymptotically increases from zero to a maximum (Fig 5A) and shows no saturation before R_n_ increases to 600 W m^-2^. GPP increases as T_air_ increases to approximately 29°C; GPP then declines slightly, despite T_air_ increasing further (Fig 5B). Likewise, the increase of VPD at the range of 0–22 hPa causes GPP increase and the GPP reaches the maximum when VPD reaches 22 hPa; GPP then declines as VPD increases to more than 22 hPa (Fig 5C). The estimated maximal values for the correlation of GPP to R_n_, T_air_ and VPD are 0.72, 0.7 and 0.74 g C m^-2^ half-hourly^-1^, respectively (Table 1).

**Table 1:** Parameters of the responses of the GPP against environmental factors.

	GPP to R_n_		GPP to T_air_				GPP to VPD			
Wulanwusu	GPP_max_	b	GPP_max_	K_1_	K_2_	K_3_	GPP_max_	K_1_	K_2_	K_3_
	0.72	22	0.70	97.43	18000	29	0.74	1.1	419.17	22

GPP_max_ is the saturation point of GPP; b is the rate of GPP; k_1_ and k_2_ are the dispersion parameters; and k_3_ is the optimal value of VPD or T_air._

**Fig 6:**
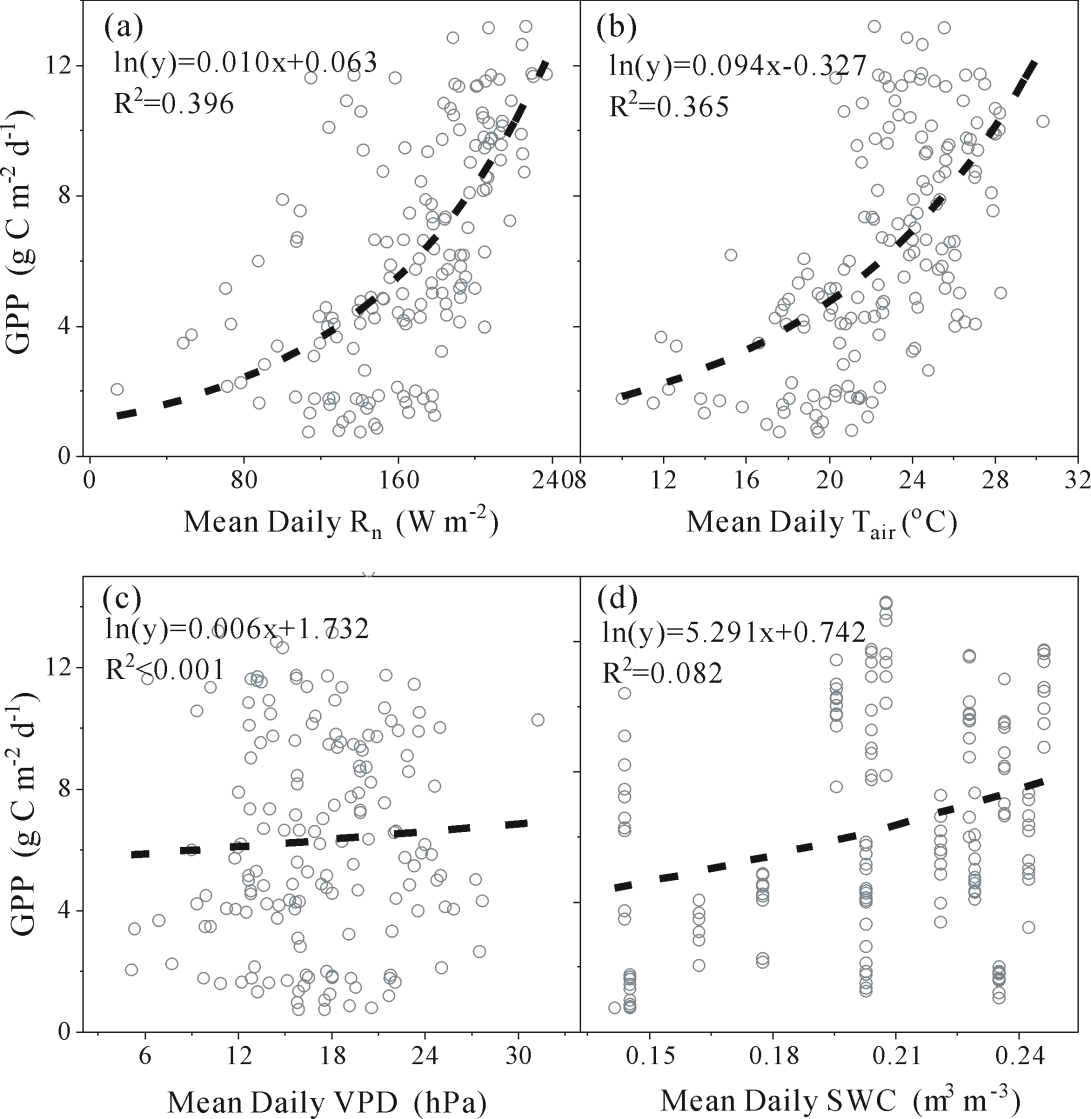
The relationship between total daily GPP (gross primary production) and mean daily R_n_ (net solar radiation), T_air_ (air temperature), VPD (vapor pressure deficit) and SWC (soil water content), respectively.

Fig 6 shows the responses of the total daily GPP to mean daily environmental variables (R_n_, T_air_, SWC and VPD), using a logistic function (ln(*y*) = a*x* + b) for this site.

At the daily time scale, the responses of total daily GPP to mean daily T_air_ and R_n_ are similar, but total daily GPP shows a stronger correlation with mean daily R_n_ (R^2^ = 0.396) than mean daily T_air_ (R^2^ = 0.37), which are more strongly correlated with total daily GPP than mean daily SWC (R^2^ = 0.08). Nevertheless, mean daily VPD is weakly correlated with total daily GPP (R^2^<0.001).

**Fig 7:**
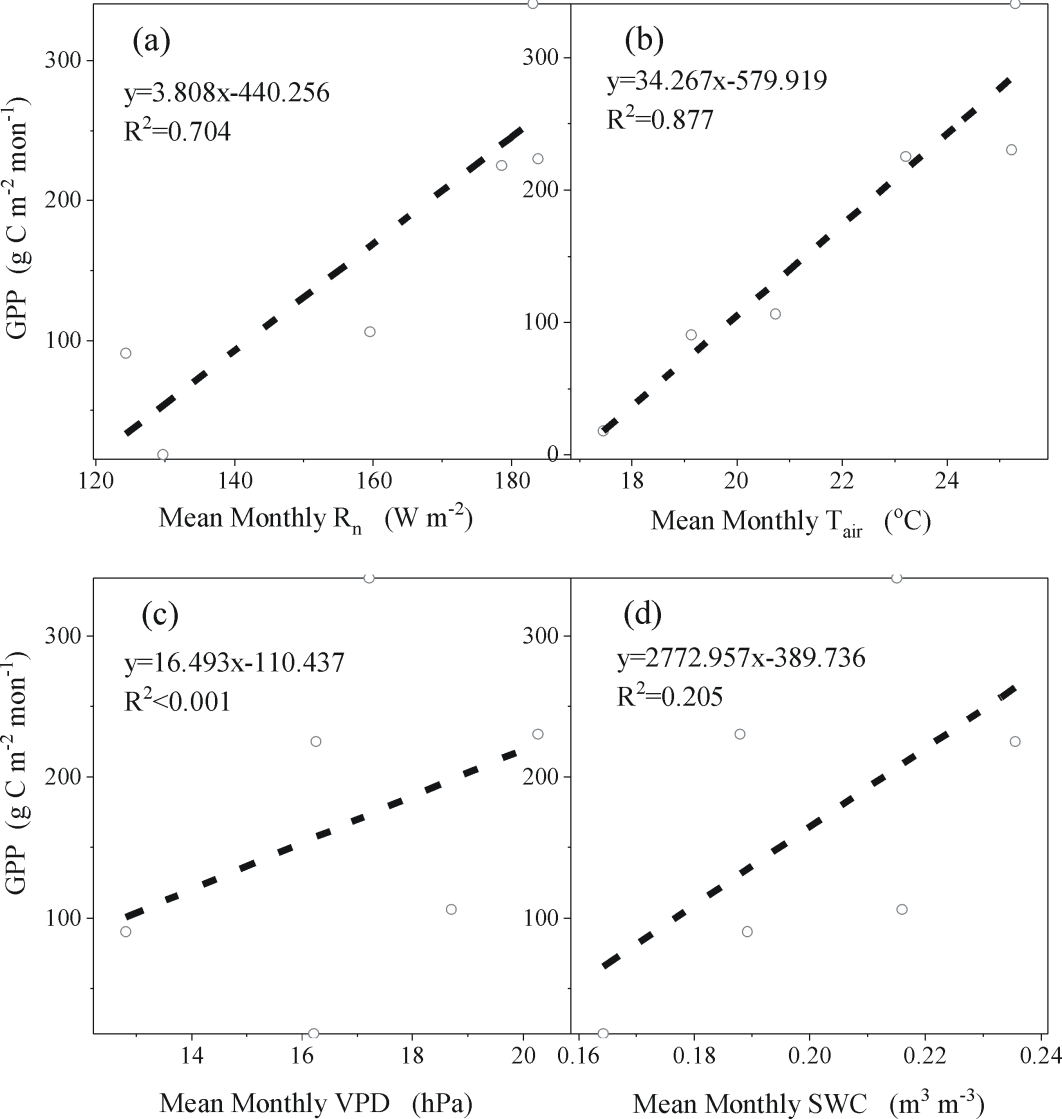
The relationship between total monthly GPP (gross primary production) and mean monthly R_n_ (net solar radiation), T_air_ (air temperature), VPD (vapor pressure deficit) and SWC (soil water content), respectively.

At the monthly time scale (Fig 7), using a linear function, total monthly GPP is more strongly correlated with mean monthly T_air_ (R^2^ = 0.88) than mean monthly R_n_ (R^2^ = 0.70), and total monthly GPP is more weakly correlated with mean monthly SWC (R^2^ = 0.21) than mean monthly T_air_ and R_n_. Mean monthly VPD, however, is still poorly correlated with total monthly GPP (R^2^<0.001). Hence, the total monthly GPP is most sensitive to mean monthly T_air_ among these environmental factors.

**Fig 8:**
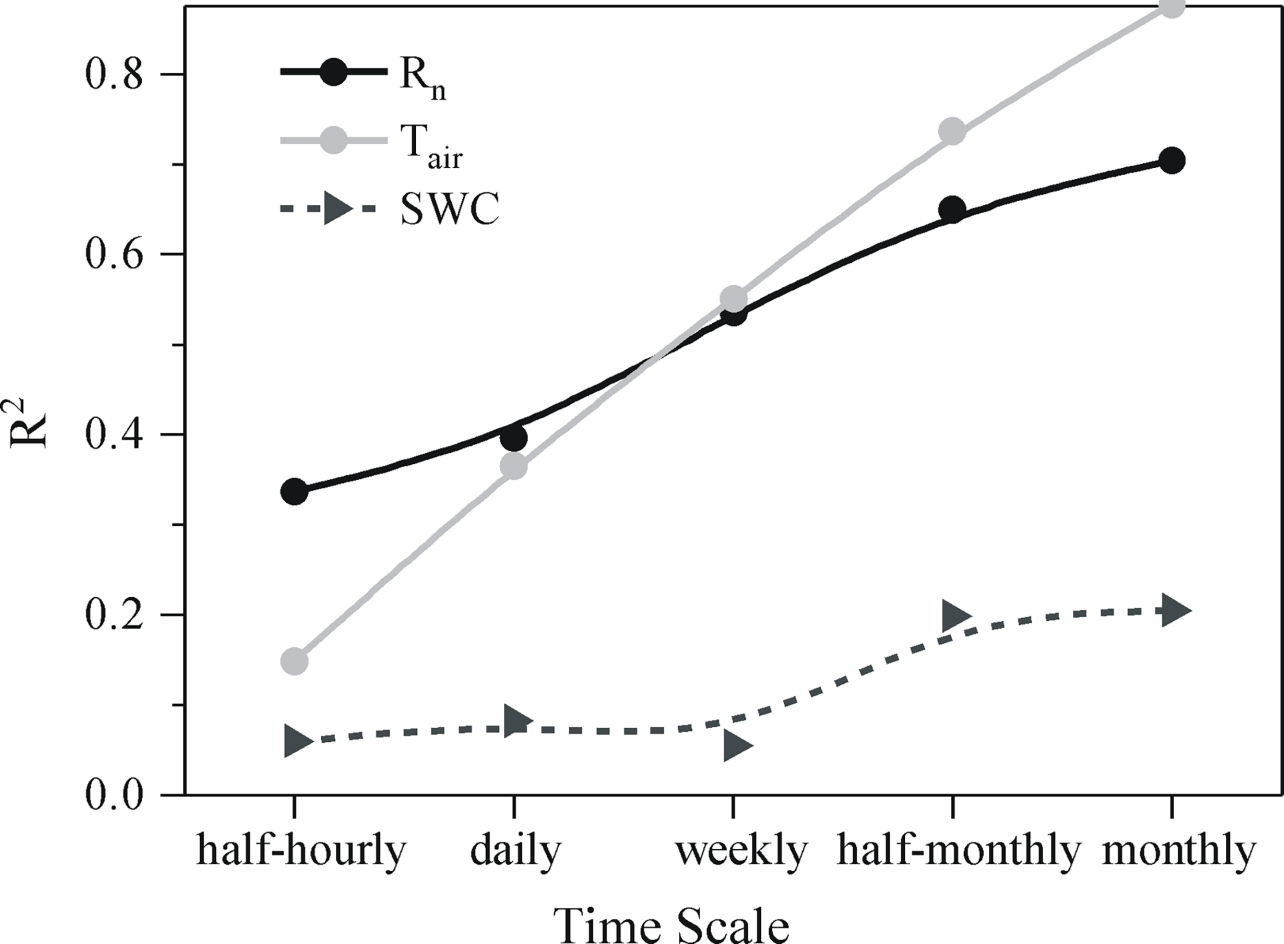
Comparisons of correlations of GPP (gross primary production) to R_n_ (net solar radiation), T_air_ (air temperature) and SWC (soil water content) at various time scales.

Fig 8 shows that GPP is more strongly correlated with T_air_ than R_n_ at time scales of half-hourly to monthly; SWC are weakly correlated with GPP at all time scales. With the scaling up of time from hour to month, the correlations of GPP to T_air_, R_n_ and SWC become stronger.

### Response of GPP residuals at the daily scale

Some researchers have contended that at the daily scale, GPP could be reliably predicted from R_n_ and T_air_ [32]. Therefore, the study simulates GPP driven separately by R_n_ and T_air_ on a daily scale. RH and VPD are strongly correlated with the GPP-R_n_ relationship (R^2^ = 0.40 and 0.17, respectively) (Table 2) and the GPP-T_air_ relationship (R^2^ = 0.46 and 0.39, respectively) (Table 3). RH is the most important variable explaining the variability of the residuals and VPD is second (Tables 2 and 3). However, SWC and Ustar have slight impacts on the GPP residuals (Tables 2 and 3). RH, Ustar, and VPD are stronger correlations to the residuals of the GPP-T_air_ relationship than the residuals of the GPP-R_n_ relationship (Tables 2 and 3).

**Table 2:** Relationships between the residuals from the regression of GPP-R_n_ and environmental variables at the daily time scale.

Variable	Regression equation	R^2^
RH	Residuals = 0.140RH-5.91987	0.402
Ustar	Residuals = -67.142Ustar^2^+21.762Ustar-1.47297	0.016
VPD	Residuals = -11.004log^2^(VPD)+29.911log(VPD)-20.148	0.171
SWC	Residuals = 16.640SWC-3.382	0.032

**Table 3:** Relationships between the residuals from the regression of GPP-T_air_ and environmental variables at the daily time scale.

Variable	Regression equation	R^2^
RH	Residuals = 0.153RH-6.554	0.459
Ustar	Residuals = -78.215Ustar^2^+23.694Ustar-1.496	0.030
VPD	Residuals = -19.991log^2^(VPD)+37.148log33-15.403	0.214
SWC	Residuals = 19.471SWC-4.037	0.043

## Discussion

### Energy balance closure

Energy balance closure (EBC) is an indicator of quality evaluation for the dataset from EC system [55]. In this study, the energy balance is not closed. The main reasons are neglected energy sinks and horizontal or vertical advection of heat and water vapor, induced by plastic film mulch, which has a great impact on the process of energy partitioning at the soil surface under the plastic film. Plastic film mulch can clearly slow the rates of soil drying; hence, soil under plastic film mulch can maintain more water than bare soil. In the cotton field, bare areas draw water not only vertically from deeper soil layers, but also horizontally from the mulched areas, which act as a “sink” for soil water. The temperature of bare soil is higher than that of mulched soil; therefore, bare areas can act as a heat source for nearby mulched areas [18]. Moreover, condensation on the inside plastic film mulch also occurs because T_air_ above plastic film mulch is higher than under mulch. In consequence, energy transfer in the soil-mulch-atmosphere system is difficult to be acquired by the EC instrument [8, 17, 19], resulting in the non-closure of energy balance. Table 4 shows the energy closure ratios off our typical arid eco-system experimental stations in central Asia. Energy closure ratio in mulched fields is 0.57, clearly less than that in non-mulched fields (CN-FUk, KZ-Ara and KZ-Bal are 0.86, 0.76 and 0.95, respectively) [56]. Therefore, energy balance closure in mulched fields is poorer than that in non-mulched fields. The comparisons further verify that energy balance nonclosure of this study is principally induced by plastic film mulch.

**Table 4:** Energy closure ratio comparsions of mulched cotton in the Wulanwusu and non-mulched vegetations in other areas.

Experimental station	Main vegetation type	Year(month)	Energyclosure ratio^4^	Mulch or non-mulched	Source
Wulanwusu	Cotton	2012(4–10)	0.57	yes	This study
CN-Fuk^1^	*Tamarix ramosissima*	2009(4–10)	0.86	no	Wang
KZ-Ara^2^	*Halostachys caspica and Tamarix chinensis*	2012(5–8)	0.76	no	Wang
KZ-Bal^3^	grassland	2012(5–9)	0.95	no	Wang

^1^CN-Fuk (Fukang Desert Ecosystem Observation and Experiment Station, Chinese Academy of Sciences) is located in Xinjiang, China.

^2^ KZ-Ara is located in Aral Lake, Kazakhstan

^3^KZ-Bal is located in Balkhash Lake, Kazakhstan

^4^Energy closure ratio (slope of LE+H against R_n_-G) represents the degree of energy balance closure.

Additionally, variations of leaf area index influence energy partition [57], thereby impacting energy balance closure. A large LAI increases transpiration, which could lessen the vertical water and heat transfer under the film mulch. In the period of flowering and boll forming, LAI is largest, but energy balance closure is slightly worse than in other stages (Table 5). The reason for this phenomenon is that the interaction of high temperature and stronger solar radiation results in the increasing of VPD and stomata closure to reduce canopy transpiration, thereby increasing the vertical water and heat transfer under the film mulch and then decreasing the energy closure ratio[57].

**Table 5:** LAI comparison for each of the growth stages.

	N	R^2^	Slope	LAI	Julian day
Sowing seed	527	0.919	0.569		108–118
Seed emergence	1680	0.906	0.578	0.3	119–153
Squaring	1248	0.920	0.607	0.5	154–179
Flowering and boll formation	3024	0.906	0.553	2.3	180–242
				3.7	
				4.5	
Boll opening	1200	0.946	0.606	4	243–267
				2.3	

N represents the number of records of carbon flux.

### Variations of GPP, R_eco_ and NEE

The cotton field is regarded as a carbon sink in all growing seasons. Compared with the study of Li et al. [14], the absolute value of the cumulative NEE in the present study is slightly higher than in the non-mulched cotton field without dripped irrigation. Also, Cuello*et al*. [12] also concluded that plastic film mulching increased grain productivity by 8–33% over non-mulching. Plastic mulching can improve soil temperature and SWC, enhance water use efficiency, increase soil microbial activity and the mineralization process, and so on, resulting in increased carbon fixation and nutrients uptake [12, 18, 58–63]. Improving soil temperature and water promotes the growth of crop roots and improves the root distribution across the soil profile [64]. These improvements enhance the ability of crops to extract more nutrients and water from soil [64].

The GPP of terrestrial vegetation is an important variable in determining the global carbon cycle, as well as interannual variation in the atmospheric carbon concentration [65]. Moreover, the responses of GPP to environmental factors are similar to the responses of NEE or R_eco_ [66]_._ More importantly, GPP is the acquisition of net carbon by photosynthesis, the process by which carbon and energy enter an ecosystem, and the best measure of the carbon that enters the ecosystem [66]. Therefore, the study uses GPP to analyze the responses of carbon fluxes to environmental factors (R_n_, T_air_, VPD, SWC and Ustar).

### Responses of GPP at half-hourly, daily and monthly time scales

At the half-hourly time scale, on the condition that other environmental factors are optimal, the boundary curves show the response of the dependent variable (GPP) to an independent variable (R_n_, T_air_ or VPD) and can describe the relations of GPP to a single meteorological factor. For instance, at low levels of R_n_, the energy supply limits GPP, but at high levels of R_n_, other factors (especially SWC and the hydraulic conductance of soil and plant) limit the GPP. When R_n_ reaches 600W m^-2^, photosynthesis reaches saturation due to the limited ability of light-harvesting; at extremely high levels of R_n_, photosynthesis decreases due to photo oxidation of photosynthetic enzymes and pigments.

In arid regions, soil moisture, to a large extent, relies on T_air_ [34]. Because plastic mulching retains the soil moisture and the cotton is regularly irrigated, soil moisture is not the limiting factor for the entire growth season [8]. Furthermore, the increase of VPD induces an increase of the evaporative demand [54] and stomatal limitation to an increase in carbon fixation in the range of 0–22 hPa. However, the decrease of stomata conductance does not induce a decrease of C fixation in the range of 0–22 hPa due to the increasing of water use efficiency of photosynthesis. When VPD > 22hPa, the increase of VPD induces an increase of evaporative demand. When the evaporative demand can not be met, the stomata close and flux rates decline to match the water constraint [53]. Nevertheless, the optimal VPD (22hPa) in a drip-irrigated, film-mulched cotton field is higher than in wet–dry–tropical savanna (10-20hPa) without film mulching and drip-irrigation [67].

Under high soil water potential, carbon fixation does not respond to an increase in VPD [68]; under moderate and severe water stress, carbon fixation is significantly reduced with increasing VPD [69]. Film mulch decreases water stress, so the optimal VPD corresponding with the maximum GPP is high in this study. Bai *et al*. [51] used 4-year data (2009, 2010, 2012 and 2013) at the same station and found that film mulched drip irrigation effected the relationship between GPP and environmental factors, such as R_n_, T_air_ and VPD.

The correlations of GPP to T_air_, R_n_ and SWC become stronger with time scaling up from half-hour to month (Fig 8). From the perspective of ecology, an ecosystem has the characteristics of resistance and resilience. Resilience is a measure of robustness and buffering capacity of the ecosystem to changing conditions [70]. Plant photosynthesis and growth maintain high resilience in the face of diurnal and seasonal variations [66]. Additionally, plastic film mulch can enhance ecosystem resistance because plastic film mulch can reduce soil erosion and control weeds [12]. Ona short time scale, the fluctuation of GPP is large in the face of changes toR_n_, T_air_ and SWC because GPP immediately responds to these perturbations; ona long time scale, GPP tends to be stable due to ecosystem resistance and resilience. Therefore, the data of GPP at a short time scale are more discrete than at a long time scale, resulting in a stronger correlation at a longertime scale. From a statistical point of view, a longertime scale can filter out some outliers of GPP and climate data and make data deviations diminish. However, the correlation of VPD to GPP is especially weak at all time scales in this region. The reason is that the protection of plastic film mulch and regular irrigation allow soil maintain sufficient water [8], and with sufficient soil water, VPD has little effect on photosynthesis [71].

Ona short time scale, the correlation of GPP to R_n_ is more sensitive than to T_air_; ona long time scale, it reverses (Fig 8). This phenomenon is determined by the biochemical characteristics of photosynthesis [66]. The most conspicuous characteristics of carbon-fixation reaction are that: photosynthetic enzymes need a great amount of nitrogen; carbon fixation depends on the products of the light-harvesting reaction (ATP and NADPH) which, in turn, depends on solar radiation; and carbon fixation is restricted by the supply of carbon dioxide [66]. The chloroplasts of leaves respond to the change of available light in a few minutes [66]. However, it takes a few days or weeks for the changes of the density of light-harvesting pigment and photosynthetic enzymesto take effect. Moreover, temperature has a great effect on the content of plant nitrogen [72]; high temperature enhances the potential of soil nitrfication and increases the openness of the soil nitrogen cycle [73]. Plastic film mulching can also improve nitrogen availability for a plant increasing the density of photosynthetic enzymes gradually. Therefore, radiation and the supply of carbon dioxide are extremely sensitive to this process at time scales of milliseconds to minutes; T_air_ is sensitive to this process at time scale of days to weeks.

However, SWC is more weaklycorrelated with GPP than R_n_ and T_air_ at all time scales. Plastic film mulching blocks parts of energy transfer from soil to air and diminishes the impact of changes of SWC on T_air_ by soil thermal characteristics, resulting in weekly correlation of SWC to the GPP residuals. At the same time, deep roots of cotton in arid region extract waterfrom the capillary fringe of the water table, and stored water in the stem can ease the supply and demand imbalance of water [66]. Plastic film mulch can also improve soil water ifthe cotton is regularly irrigated. Therefore, providing enough soil water with cotton scarcely induces the decreasing of stomatal conductance so that GPP is unresponsive to SWC [67].

Ustar is also poorly related with GPP, as shown in Tables 2 and 3. Plastic film mulching reduces Ustar, and hence the rate of wind on the surface increases. Wind can accelerate the diffusion of carbon dioxide from the atmosphere to leaves, resulting in the increasing of carbon fixation, but strong light in arid region increases stomatal conductance; then, the limitation of the density of carbon dioxide to photosynthesis is reduced to a minimum, and Ustar and wind decrease the GPP residuals.

From the perspective of resource allocation, envirommental changes, such as the fluctuation of nutrient supply and storms, vary the relative abundance of resources. Therefore, it is inevitable that there aredifferent limiting factors for carbon fluxes at different time scales [66].

## Conclusions

Drip irrigation under plastic mulch is widely used in Xinjiang, the largest arid region in China. This study investigated the relationships between carbon fluxes and environmental factors in a drip-irrigated, film-mulched cotton field of this water-limited region. The cotton field is a strong sink of carbon, and its cumulative NEE over the entire growing season is -304.8 g C m^-2^. The cumulative NEE in mulched cotton field is higher than that in non-mulched cotton field. RH, R_n_, and T_air_ are important controlling factors of carbon balance, RH was the most limiting environmental factor. However, SWC and Ustar have slight impacts on the GPP residuals. Additionally, RH, Ustar and VPD are slightly more correlated with the simulated GPP driven by T_air_ than by R_n_. RH, VPD and Ustar are more sensitive to T_air_ than R_n_ [66]. High temperature decreases RH and increases VPD. Decreasing of Ustar increases the rate of wind and evaporation, which decreases leaf temperature [66]. In general, the total daily GPP could be reliably predicted from R_n_ and T_air_; at the same time, RH, Ustar, SWC and VPD are also important factors to constrain the response of GPP to R_n_ and T_air_ at different degrees of limitation.

## Supporting information

S1 File The minimal data set. This file includes the data of Fig 1–7.
(XLSX)
